# Knowledge, Attitude, Awareness, and Practice Regarding the Use of Interdental Aids Among Dental Professionals and Students: A Cross-Sectional Study

**DOI:** 10.7759/cureus.89669

**Published:** 2025-08-09

**Authors:** Huda H Mohamed, Hana M Ghaith, Sara Mohamed A. M Bogazia

**Affiliations:** 1 Department of Periodontology, Faculty of Dentistry, University of Benghazi, Benghazi, LBY; 2 Department of Oral Biology, Ajdabiya University, Ajdabiya, LBY

**Keywords:** attitude, dental professionals, dental students, interdental cleaning aids, knowledge, oral hygiene

## Abstract

Introduction: Interdental aids are necessary to effectively clean between the teeth by removing biofilms. Students and professionals play a crucial role in oral health promotion. Their attitude, knowledge, and behavior indicate how well they take care of their teeth and motivate patients to follow good oral health habits. The aim of this study was to assess dental professionals' and students' knowledge, attitude, awareness, and practice regarding the use of interdental aids and also to identify whether dental education helps to increase their knowledge, attitude, and practice about the use of interdental aids.

Methods: An online survey using a 15-item questionnaire with four sections was carried out. This was performed using Google Forms to assess the knowledge and use of interdental aids by 237 Libyan dental students and professionals.

Results: The respondents included 174 female respondents (73.4%), and 127 of them (53.6%) were aged between 20 and 30 years. Most participants were practicing in Benghazi (152; 64.1%) and represented all educational levels, from first-year students to PhD graduates. A total of 225 subjects (94.9%) knew about interdental aids. The specialist and postgraduate groups showed significantly higher identification of health benefits compared to the younger groups (p = 0.005).

Conclusions: Overall awareness and knowledge of interdental aids were high and increased with the level of education but also had gaps, especially among less experienced trainees. During dental education and professional development, special education programs can promote the use of standardized and efficient interdental hygiene procedures and improve the oral health of their patients.

## Introduction

Dental biofilm accumulation, caused by poor oral hygiene practices, is the primary etiologic factor of gingivitis and periodontitis, which are the most common diseases of the oral cavity. They not only impair teeth and periodontal tissues but also the overall health [1]. Fortunately, these diseases are preventable through a combination of professional care and daily oral hygiene practices via mechanical and chemical methods [2]. There is evidence to support that the people who adopt good oral hygiene are less likely to develop periodontal disease and retain their teeth [3]. 

The mechanical brushing of teeth is considered the most effective procedure for plaque control [4]. It is advised that effective brushing should be done at least twice daily, taking approximately two minutes [5]. However, tooth brushing alone incompletely cleans the interproximal areas, as they are not easily accessible with a toothbrush; therefore, for effective removal of the residual interproximal plaque and achieving optimal interdental hygiene, additional interdental aids are used to overcome the limitations of tooth brushing [6,7]. These are dental floss, interdental brushes, single-tufted brushes, and rubber-tip stimulators [8]. Clinically, the development and establishment of gingivitis and periodontitis tend to be more rapid in interdental areas than on facial and lingual surfaces [9]. This is because there is interdental gingiva between teeth just below the contact point, which creates a secluded place that cannot be reached easily; therefore, interdental cleaning is important during the treatment and maintenance of gingivitis and periodontitis cases, but most patients use interdental aids inappropriately. Dentists and dental students must be educated and motivate patients to use interdental tools effectively, so they can have good knowledge and habits themselves [10]. However, there is limited research assessing the awareness and use of interdental aids among dental professionals and students, particularly in Libya. This gap in knowledge needs to be addressed to help improve dental education and ultimately enhance patient care.

The purpose of the present study is: (i) to assess dental professionals' and students' knowledge, attitudes, awareness and practice regarding the use of interdental aids and (ii) to determine whether different levels of dental education and professional experience influence the knowledge, attitudes, and practices of dental students and professionals regarding the use of interdental aids.

## Materials and methods

This cross-sectional study was carried out on dental students who are currently enrolled in Bachelor of Dental Surgery (B.D.S.) programs and professionals who hold a B.D.S. degree or postgraduate qualifications (Master’s, PhD, or specialty training) to evaluate their knowledge, attitude, awareness, and practice of interdental aids. All procedures were in accordance with ethical principles, and the study protocol was approved by the Faculty of Dentistry, University of Benghazi Scientific Research Ethics Committee in April 2025 [Reference Code: 0276] before data collection.

Inclusion criteria

Participants included undergraduate dental students, graduates holding a B.D.S. degree, and postgraduate dental specialists practicing within Libya.

Exclusion criteria

Individuals excluded from the study were non-dental participants, those who submitted incomplete responses, and dental professionals practicing outside Libya.

Participants were recruited via convenience sampling among staff, clinics, and student cohorts at multiple Libyan dental schools and clinics. The study population was divided into four groups based on the year of study as follows: Group I (Preclinical): first and second-year students, Group II (Clinical students): third, fourth year, and internship students, Group III: General practitioners and B.D.S. graduates (Group III), and Group IV: Specialists and Postgraduate group.

Instrument and data collection

A structured questionnaire was developed based on earlier studies and guidelines about oral hygiene and interdental aids [9,11-14]. Although we did not use a formal validation tool, three senior experts reviewed the questionnaire to ensure the questions were clear and relevant. We also tested it with a small group, which confirmed that the questions were easy to understand. The questionnaire was then administered online using Google Forms. All participants completed the self-administered questionnaire.

The questionnaire started with demographic questions about age, gender, education, years of experience, and field of practice. Then, it included four sections: Knowledge (four questions), Awareness (three questions), Attitude (three questions), and Practice (five questions). It was 15 different types of questions like Yes/No, multiple choice, frequency, Likert scale, and open-ended to get a complete understanding of the participants’ views.

Statistical analysis

Frequencies and percentages were computed for all demographic and questionnaire items. Results were tabulated to summarize participant characteristics and response distributions. Two distinct comparisons were made: Preclinical vs. Postclinical Students (n = 98) and General Practitioners vs. Specialists (n = 139)

For each comparison (Preclinical vs. Postclinical; General Practitioners vs. Specialists), 2 × 2 (or larger) contingency tables were constructed for each question. Pearson’s chi-square test was performed at α = 0.05 to assess group differences. Following chi-square testing, post hoc power analyses were conducted with G*Power 3.1 (Heinrich-Heine-Universität Düsseldorf, Düsseldorf, Germany) to ensure statistical adequacy. Data extraction and cleaning were performed using Microsoft Excel (Microsoft Corporation, Redmond, USA). Statistical analyses (chi-square) were run using IBM SPSS Statistics for Windows, Version 25 (Released 2017; IBM Corp., Armonk, New York, United States).

## Results

A total of 237 dental practitioners participated in this survey. Most participants were female (n = 174, 73.4%) and aged between 20 and 30 years (n = 127, 53.6%). The majority practiced in Benghazi (n = 152, 64.1%) and were affiliated with the University of Benghazi (n = 143, 60.3%). Participants included both undergraduate dental students and professionals. Detailed demographic distributions, including education level, years of experience, and specialty, are presented in Table 1.

**Table 1:** Demographic Profiles of Study Participants (N = 237) UOO, University of Omar AL-mokhtar; LIMU, Libyan International Medical university; UOA, University of Ajdabiya; UOB, University of Benghazi; UOS, University of Sebha; UOT, University of Tripoli; GP, General Practitioner; UG, Undergraduate.

Variable	Category	N (%)
Gender	Female	174 (73.4)
	Male	63 (26.6)
Age categories	20-30	127 (53.6)
	31-40	54 (22.8)
	41-50	49 (20.7)
	51-60	7 (3.0)
City	Ajdabiya	13 (5.5)
	Al Bayda	26 (11.0)
	Benghazi	152 (64.1)
	Derna	7 (3.0)
	Sebha	10 (4.2)
	Shahat	6 (2.5)
	Toubruk	5 (2.1)
	Tripoli	18 (7.6)
University	Other	7 (3.0)
	UOO	31 (13.1)
	Balagrea	6 (2.5)
	LIMU	22 (9.3)
	UOA	13 (5.5)
	UOB	143 (60.3)
	UOS	8 (3.4)
	UOT	7 (3.0)
Education	UG 1st–3rd yr.	14 (5.9)
	UG 4th–Intern	84 (35.4)
	Graduate (BDS)	58 (24.5)
	Postgrad (MDS)	66 (27.8)
	Postgrad (PhD)	15 (6.3)
Experience	1–5 years	55 (23.2)
	5–10 years	21 (8.9)
	More than 10 years	85 (35.9)
	None	76 (32.1)
Academic level and Specialty	Student	30 (12.7)
	Intern	6 (2.5)
	Gp	115 (48.5)
	Basic	10 (4.2)
	Dental public health	7 (3.0)
	Oral and maxillofacial surgery	8 (3.4)
	Oral Medicine	4 (1.7)
	Orthodontics	6 (2.5)
	Pedodontics	12 (5.1)
	Periodontists	15 (6.3)
	Prosthetic	14 (5.9)
	Restorative	10 (4.2)

Knowledge

Overall, 225 (94.9%) of the respondents knew about interdental aids, and 234 (98.7%) correctly defined their main purpose. Interdental brushes (185; 78.1%) and dental floss (221; 93.2%) were the best-known aids, and 203 (85.7%) knew that they must be used on a daily basis, as shown in Table 2.

**Table 2:** Knowledge of Interdental Aid Use Among Participants (N = 237)

Question	Response	n	%
Q1. Have you heard about interdental aids?	Yes	225	94.9
	No	12	5.1
Q2. What are interdental aids primarily used for?	Cleaning between teeth	234	98.7
	Strengthening enamel	1	0.4
	Whitening teeth	1	0.4
	Other	1	0.4
Q3. Which of the following are examples of interdental aids? (select all that apply)	Dental floss	221	93.2
	Interdental brushes	185	78.1
	Toothpicks	145	61.2
	Water floss	139	58.6
	Multiple interdental aids	180	75.9
Q4. How often should interdental aids be used according to dental professionals?	Daily	203	85.7
	Occasionally	14	5.9
	Weekly	13	5.5
	Not sure	7	3.0

According to students, pre-clinical and post-clinical students showed similar overall knowledge about interdental aids. However, post-clinical students were much more likely to know their correct purpose - 83 (86.5%) compared to just 13 (13.5%) among pre-clinical students (p = 0.045). There were no clear differences between the two groups in identifying types of interdental aids, or in knowing how often these aids should be used (p > 0.05) (Table 3).

**Table 3:** Comparison of Knowledge of Interdental Aids by Training Stage and Professional Status

Question	Response	Preclinical (n, %)	Post-clinical (n, %)	p-value
Have you heard about interdental aids?	Yes	13 (14.1%)	79 (85.9%)	0.863
	No	1 (16.7%)	5 (83.3%)	
What are interdental aids primarily used for?	Cleaning between teeth	13 (13.5%)	83 (86.5%)	0.045
	Strengthening enamel	1 (100.0%)	0 (0.0%)	
	Other	0 (0.0%)	1 (100.0%)	
Examples of interdental aids:	Dental Floss	13 (14.6%)	76 (85.4%)	0.775
	Interdental brush	7 (10.8%)	58 (89.2%)	0.163
	Toothpicks	7 (12.5%)	49 (87.5%)	0.560
	Water floss	8 (12.9%)	54 (87.1%)	0.608
	Multiple aids	5 (10.6%)	42 (89.4%)	0.322
How often should interdental aids be used according to dental professionals?	Daily	10 (12.5%)	70 (87.5%)	0.073
	Weekly	1 (14.3%)	6 (85.7%)	
	Occasionally	1 (12.5%)	7 (87.5%)	
	Not sure	2 (66.7%)	1 (33.3%)	
Question	Response	Graduate (BDS)	specialist	p-value
q1 Have you heard about interdental aids?	Yes	54 (93.1 %)	79 (97.5 %)	0.205
	No	4 (6.9 %)	2 (2.5 %)	
q2 What are interdental aids primarily used for?	Cleaning between teeth	57 (98.3 %)	81 (100.0 %)	0.236
	Whitening teeth	1 (1.7 %)	0 (0.0 %)	
q3 Which of the following are examples of interdental aids?	Dental floss	54 (93.1%)	78 (96.3%)	320
	Interdental brushes	48 82.8%	72 88.9%	0.215
	Toothpicks	36 62.1%	53 65.4%	0.409
	Water floss	47 81.0%	71 87.7%	0.201
q4 How often should interdental aids be used according to dental professionals?	Weekly	3 (5.2 %)	3 (3.7 %)	0.528
	Occasionally	2 (3.4 %)	4 (4.9 %)	
	Daily	50 (86.2 %)	73 (90.1 %)	
	Not sure	3 (5.2 %)	1 (1.2 %)	

According to professional status,* *general practitioners and specialists both showed high levels of knowledge - 54 (93.1%) vs. 79 (97.5%) (p = 0.205) - and understanding of the purpose of interdental aids - 57 (98.3%) vs. 81 (100.0%) (p = 0.236) as shown in Table 3. Most participants in both groups supported daily use of these aids, and there were no significant differences in recognizing different types or in their attitudes toward using them (p > 0.05).

Awareness

Overall, 97.9% of participants (232 out of 237) were aware of the health benefits of interdental aids (Table 4), and the same proportion agreed that cleaning between the teeth is just as important as brushing (Table 4). However, only 59.1% (140 out of 237) reported receiving formal training or education on their use.

**Table 4:** Awareness about Interdental Aid Use Among Participants (N = 237)

Question	Response	n	%
Q5. Are you aware of the health benefits of using interdental aids?	Yes	232	97.9
	No	5	2.1
Q6. Do you think interdental cleaning is as important as brushing your teeth?	Yes	232	97.9
	No	5	2.1
Q7. Have you received any formal education or training about the use of interdental aids?	Yes	140	59.1
	No	97	40.9

According to students, post-clinical students were far more likely to know about the health benefits of interdental aids compared to preclinical students (87.5% vs. 12.5%, p < 0.001). No significant differences were noted for perceived value of interdental cleaning (85.1% vs. 14.9%, p = 0.404) or rates of formal training (89.6% vs. 10.4%, p = 0.110) (Table 5), and so awareness and receipt of teaching build up more slowly across the curriculum.

**Table 5:** Comparison of Interdental Aid Awareness and Training by Education Level and Professional Status

Question	Response	Preclinical (n, %)	Post-clinical (n, %)	p-value
Aware of health benefits of interdental aids?	Yes	12 (12.5%)	84 (87.5%)	<0.001
	No	2 (100.0%)	0 (0.0%)	
Interdental cleaning as important as brushing?	Yes	14 (14.9%)	80 (85.1%)	0.404
	No	0 (0.0%)	4 (100.0%)	
Received formal education or training?	Yes	7 (10.4%)	60 (89.6%)	0.11
	No	7 (22.6%)	24 (77.4%)	
Question	Response	Graduate (BDS)	Specialist	p-value
Are you aware of the health benefits of interdental aids?	Yes	57 (98.3%)	79 (97.5%)	0.766
	No	1 (1.7%)	2 (2.5%)	
Do you think interdental cleaning is as important as brushing your teeth?	Yes	58 (100.0%)	80 (98.8%)	0.396
	No	0 (0.0%)	1 (1.2%)	
Have you received formal education/training on interdental aids? (Q7)	Yes	33 (56.9%)	40 (49.4%)	0.382
	No	25 (43.1%)	41 (50.6%)	

According to professional status, in a comparison between the specialists and the general practitioners, the two were found to have equally high awareness: (98.3% vs. 97.5%, p = 0.766) respondents recognized the advantages, and nearly all were in agreement on its importance (100% vs. 98.8%, p = 0.396). Rates of formal training were similar (56.9% vs. 49.4%, p = 0.382), indicating that education background on interdental aids remains low in clinical practice settings (Table 5).

Attitudes

Overall, the majority of responders had positive attitudes towards interdental aids: 191 (80.6%) thought their use was "very important" for oral health, and 45 (19.0%) found them "somewhat important." Two hundred and twenty-three (94.1%) responders would recommend interdental aids to family or friends, with only 14 (6.0%) unsure or opposed (Table 6). Regarding the challenges that prevent the use of interdental aids, 139 (58.6%) mentioned lack of time, 60 (25.3%) difficulty in use, 47 (19.8%) cost, 43 (18.1%) lack of knowledge or other reasons, and 78 (32.9%) reported more than one challenge.

**Table 6:** Attitude toward Interdental Aid Use Among Participants (N = 237)

Question	Response	n	%
How important do you think it is to use interdental aids for oral health?	Very important	191	80.6
	Somewhat important	45	19
	Not important	1	0.4
Would you recommend interdental aids to friends or family members?	Yes	223	94.1
	No	7	3
	Not sure	7	3
What prevents you from using interdental aids?	Time barrier	139	58.6
	Difficulty in use	60	25.3
	Cost	47	19.8
	Lack of knowledge/other	43	18.1
	Multiple reasons	78	32.9

According to students, preclinical and post-clinical students showed similarly strong attitudes: 62 (86.1%) post-clinical versus 10 (13.9%) preclinical students considered interdental aids "very important" (p = 0.889), and 76 (86.4%) vs. 12 (13.6%) said they would "recommend them" (p = 0.805). Regarding the challenges that prevent the use of interdental aids, the only significant difference was "lack of knowledge," reported by seven (25.9%) preclinical students compared to 20 (74.1%) post-clinical students (p = 0.025). Other challenges were similar across all training stages (Table 7).

**Table 7:** Comparison of Attitudes and Perceived Barriers to Interdental Aid Use by Training Stage and Professional Status

Question	Response	Preclinical (n, %)	Post-clinical (n, %)	p-value
Importance of interdental aids	Very important	10 (13.9%)	62 (86.1%)	0.889
	Somewhat important	4 (16.0%)	21 (84.0%)	
	Not important	0 (0.0%)	1 (100.0%)	
Recommend to friends/family?	Yes	12 (13.6%)	76 (86.4%)	0.805
	No	1 (16.7%)	5 (83.3%)	
	Not sure	1 (25.0%)	3 (75.0%)	
What prevents you from using interdental aids?	Time	6 (10.5%)	51 (89.5%)	0.21
	Difficulty in use	5 (17.2%)	24 (82.8%)	0.588
	Lack of knowledge	7 (25.9%)	20 (74.1%)	0.025
	Cost	5 (20.8%)	19 (79.2%)	0.291
	Other reasons	2 (13.3%)	13 (86.7%)	0.909
Question	Response	Graduate (BDS)	Specialist	p-value
How important is using interdental aids for oral health?	Very important	50 (86.2%)	69 (85.2%)	0.866
	Somewhat important	8 (13.8%)	12 (14.8%)	
Would you recommend interdental aids to friends/family?	Yes	57 (98.3%)	78 (96.3%)	0.665
	No	0 (0.0%)	1 (1.2%)	
	Not sure	1 (1.7%)	2 (2.5%)	
What prevents you from using interdental aids?	Time barrier	35 (60.3%)	47 (58.0%)	0.784
	Difficulty in use	15 (25.9%)	16 (19.8%)	0.394
	Lack of knowledge	12 (21.1%)	11 (13.6%)	0.246
	Cost	9 (15.5%)	14 (17.3%)	0.782
	Other reasons	10 (17.2%)	18 (22.2%)	0.47

According to professional status,both specialists and general practitioners were equally likely to rate interdental aids as "very important" (86.2% vs. 85.2%, p = 0.866) and to prescribe them (98.3% vs. 96.3%, p = 0.665). Time pressures remained the most common challenges for both professionals (60.3% vs. 58.0%, p = 0.784), whereas other reasons like difficulty of use, cost, and others were equally distributed (all p > 0.39).

Practice

Overall, 191 out of 237 (80.6%) reported using interdental aids. Among these users, 105 (44.3%) used them daily, 79 (33.3%) several times a week, and 42 (17.7%) occasionally, while 11 (4.6%) stated they never used them. Dental floss was the most commonly used aid (178, 75.1%), followed by toothpicks and interdental brushes (both 23, 9.7%) and water flossers (9, 3.8%). Dentists were the main source of information about cleaning between teeth for 175 participants (73.8%). Other sources included online videos and articles (19, 8.0%), other unspecified sources (20, 8.4%), and social media (18, 7.6%). When asked about challenges in using interdental aids, 198 participants (83.5%) responded “yes,” indicating they faced at least one challenge (Table 8).

**Table 8:** Practice of Interdental Aid Use Among Participants (N = 237)

Question	Response	n	%
Do you currently use any interdental aids?	Yes	191	80.6
	No	46	19.4
How often do you use interdental aids?	Daily	105	44.3
	A few times a week	79	33.3
	Occasionally	42	17.7
	Never	11	4.6
What type of interdental aid do you use most often?	Dental floss	178	75.1
	Toothpicks	23	9.7
	Interdental brushes	23	9.7
	Water flossers	9	3.8
	Other	4	1.7
Where do you typically learn about oral hygiene practices?	Dentist	175	73.8
	Friends/Family	5	2.1
	Online articles/videos	19	8
	Social media	18	7.6
	Other	20	8.4
Do you face any challenges in using interdental aids?	Yes	198	83.5
	No	28	11.8
	Unspecified	11	4.6

According to students, when comparing preclinical and postclinical students, they reported similar proportions of current interdental aid use, with 11 (14.7%) among preclinical vs. 64 (85.3%) postclinical students (p = 0.846), and the frequencies of daily use: 8 (18.2%) vs. 36 (81.8%) respectively (p = 0.288) (Table 9). Dental floss was still the preferred choice, with 9 (12.2%) among preclinical students vs. 65 (87.8%) among postclinical students (p = 0.521), and dentists were commonly reported as educators, with 9 (12.5%) vs. 63 (87.5%) respectively (p = 0.192). Problems encountered did not differ significantly by training stage (p = 0.344), suggesting that practical challenges exist regardless of clinical experience.

**Table 9:** Comparison of Interdental Aid Practice by Training Stage and Professional Status

Question	Response	Preclinical (n, %)	Post-clinical (n, %)	p-value
Do you currently use interdental aids?	Yes	11 (14.7%)	64 (85.3%)	0.846
	No	3 (13.0%)	20 (87.0%)	
Frequency of use	Daily	8 (18.2%)	36 (81.8%)	0.288
	A few times/week	3 (9.1%)	30 (90.9%)	
	Occasionally	1 (6.7%)	14 (93.3%)	
	Never	2 (33.3%)	4 (66.7%)	
Type most often used	Dental floss	9 (12.2%)	65 (87.8%)	0.521
	Toothpicks	3 (30.0%)	7 (70.0%)	
	Interdental brushes	2 (20.0%)	8 (80.0%)	
	Water flossers	0 (0.0%)	2 (100.0%)	
	Other	0 (0.0%)	2 (100.0%)	
Source of oral hygiene information	Dentist	9 (12.5%)	63 (87.5%)	0.192
	Friends/Family	0 (0.0%)	3 (100.0%)	
	Online articles/videos	1 (16.7%)	5 (83.3%)	
	Social media	4 (36.4%)	7 (63.6%)	
	Other	0 (0.0%)	6 (100.0%)	
Face challenges in use?	Yes	13 (16.9%)	64 (83.1%)	0.344
	No	1 (6.7%)	14 (93.3%)	
Question	Response	Graduate (BDS)	Specialist	p-value
Do you currently use interdental aids? (Q11)	Yes	48 (82.8%)	68 (84.0%)	0.852
	No	10 (17.2%)	13 (16.0%)	
How often do you use interdental aids? (Q12)	Daily	20 (34.5%)	41 (50.6%)	0.032
	A few times a week	25 (43.1%)	21 (25.9%)	
	Occasionally	9 (15.5%)	18 (22.2%)	
	Never	4 (6.9%)	1 (1.2%)	
What type of interdental aid do you use most often? (Q13)	Dental floss	40 (69.0%)	64 (79.0%)	0.72
	Toothpicks	7 (12.1%)	6 (7.4%)	
	Interdental brushes	6 (10.3%)	7 (8.6%)	
	Water flossers	4 (6.9%)	3 (3.7%)	
	Other	1 (1.7%)	1 (1.2%)	
Where do you typically learn about oral hygiene practices? (Q14)	Dentist	47 (81.0%)	56 (69.1%)	0.123
	Friends/Family	2 (3.4%)	0 (0.0%)	
	Online articles/videos	4 (6.9%)	9 (11.1%)	
	Social media	1 (1.7%)	6 (7.4%)	
	Other	4 (6.9%)	10 (12.3%)	
Do you face challenges in using interdental aids? (Q15)	Yes	48 (82.8%)	73 (90.1%)	0.431
	No	7 (12.1 %)		

According to professional status, specialists utilized interdental care more than general practitioners 41(50.6%) vs. 34.5%, p = 0.032). This suggests that specialists will be more likely to utilize interdental care. Moreover, overall use was equal in the two groups 68(84.0%) vs. 48(82.8%). The preference for assistance (dental floss: 64(79.0%) vs. 40(69.0%), (p = 0.720), information sources (p = 0.123), and obstacle (p = 0.431) were also comparable. This shows that both specialists and general dentists find it equally necessary to clean between teeth but face the same challenges in doing so (Table 9).

## Discussion

Dental students and professionals play an important role in educating and motivating patients regarding oral health. A basic but important part of this is helping people understand how to clean between their teeth using tools like dental floss or interdental brushes. These aids are necessary because regular brushing cannot reach the spaces between teeth, and plaque buildup in these areas can lead to periodontal diseases [15]. When dental professionals have good knowledge and habits themselves, they can better guide and motivate their patients. That’s why it is important for students and professionals to be well-trained and aware of how to use these aids effectively. Thus, this study aimed to assess the knowledge, attitudes, awareness, and practices related to these aids among dental students and professionals at different stages of their education and careers [2,9]. Two hundred and thirty-seven participants engaged in the study were dental students and professionals, predominantly female (174; 73.4%) and aged 20-30. Most practiced in Benghazi 152 (64.1%) and held affiliations with the University of Benghazi 143 (60.3%). Educationally, the cohort spanned from undergraduate students (84; 35.4%) to MDS and PhD graduates (81; 34.1%), with a wide range of clinical experience, from none (76; 32.1%) to over 10 years (85; 35.9%). General practitioners comprised nearly half of the sample, 115 (48.5%), reflecting a broad cross-section of dental roles.‏

The majority of participants (225; 94.9%) had heard about interdental aids, and 234 (98.7%) correctly understood their primary function: cleaning between teeth. Dental floss and interdental brushes were recognized by over 180 (75%) of participants, while 203 (85.7%) correctly identified daily use as the recommended frequency. These findings indicate solid foundational knowledge across different educational levels.

Previous studies on interdental aids have reported similar results. For example, Koregol et al. found that 80.1% of interns and undergraduate students were aware of interdental cleaning aids and understood their role in maintaining periodontal health (89.6%) [9]. Gupta et al. (2020) also reported that knowledge and awareness improved with academic advancement. In their study, first- and second-year B.D.S. students had the lowest mean knowledge score (5.05 ± 0.29), while postgraduates and dental staff from Periodontics and Public Health Dentistry showed the highest scores (14.40 ± 0.37). Intermediate scores were reported among third- and final-year B.D.S. students (6.47 ± 1.69), interns (8.50 ± 0.67), and postgraduates from other specialties (9.16 ± 0.85) [13]. This pattern supports our findings, showing that increased education and clinical experience are associated with improved knowledge regarding interdental aids.

Nearly all participants in our study (232, 97.9%) showed high awareness of the health benefits of interdental aids and considered interdental cleaning to be as important as tooth brushing. However, only 140 (59.1%) reported having received formal education or training on the proper use of these aids. This lack of formal training was mainly observed among preclinical and early clinical students, while most general practitioners and specialists had undergone such training. This reveals a gap between general awareness and formal instruction, indicating that structured teaching on interdental aid use is still insufficiently covered in undergraduate curricula and continuing education programs.

Similarly, Koregol et al. found that (86.3%) of dental students and interns were aware that brushing alone was inadequate for cleaning interproximal areas, and 80.8% showed awareness of the purpose of interdental cleaning aids. However, only 81.0% had received information about these aids from their dentists, suggesting that formal training might not be universally available [9].

In another study involving dental professionals, comparable levels of awareness were noted: 74.5% of MDS practitioners and 25.92% of BDS practitioners demonstrated awareness of interdental cleaning objectives. Despite differences in participant groups, these findings collectively highlight a consistent pattern: while general awareness of interdental aids is relatively high, formal education and structured training remain limited [2]. Therefore, there is a clear need to reinforce formal education and practical training, especially early in dental education.

Positive attitudes prevailed, as 191 (80.6%) considered interdental aids as very important to oral health, and 223 (94.1%) would recommend their use to others. The main obstacles to personal use were lack of time (139, 58.6%), complexity of use (60, 25.3%), cost (47, 19.8%), and insufficient knowledge (43, 18.1%). Although interest is high, practical limitations still restrict regular use. Previous studies have similarly shown that most dental students and professionals recognized the importance of interdental aids in maintaining oral hygiene and frequently advised others on their use [9,14,16,17], although many felt these tools were time-consuming [9,17].

In our study, 191 (80.6%) of participants reported using interdental aids, with 105 (44.3%) using them daily and 79 (33.3%) multiple times a week. Dental floss was the most frequently used aid (178, 75.1%), and dentists were the main source of information (175, 73.8%). However, 198 (83.5%) reported facing at least one difficulty in using these aids, reflecting the challenges identified under attitudes. Our results were in accordance with Koregol et al., who reported that 80.8% of respondents were aware of interdental aid use and 74.2% used them regularly, having learned about them primarily from dentists (81.0%) [9]. In contrast, other studies found lower usage rates of interdental cleaners, especially dental floss, with social media being the main source of information [18-20].

Overall, dental professionals show excellent theoretical knowledge, awareness, and attitudes toward interdental aids. However, these strengths do not consistently lead to practical application. The disparity between high acknowledgment of benefits and reported challenges-particularly limited training and time constraints-highlights areas for enhanced educational focus and the development of more user-friendly interdental products. Addressing these gaps could promote more consistent and accessible adoption of interdental hygiene practices.

This study addresses an important gap in the literature, as there is limited research on interdental aid awareness and use across different levels of dental education, particularly in Libya. Despite this strength, the study has some limitations. The use of a self-reported questionnaire made it difficult to assess the authenticity of responses. Although the survey was conducted across several locations and universities in Libya, the sample size was relatively small, and participant engagement was limited. Future studies may benefit from larger samples and, where feasible, the use of mixed methods, including interviews, to enhance data quality.

## Conclusions

Overall, this study shows that the level of education plays a key role in how well dental students and professionals understand and use interdental aids. Higher educational levels were associated with better knowledge, attitudes, and practices regarding these preventive tools. To help students develop good oral hygiene habits early, preventive dentistry courses should be incorporated into the dental curriculum, particularly during the pre-clinical years. Continuing education for professionals is also important to reinforce and maintain these behaviors. By taking these steps, future dental professionals will be better equipped to provide effective preventive care and improve the oral health of their patients.

## Appendices

Questionnaire

*Demographic Information* 1. Age: _______________ 2. Gender: o ☐ Male o ☐ Female o ☐ Other 3. Year of Study: o ☐ 1st year o ☐ 2nd year o ☐ 3rd year o ☐ 4th year o ☐ Other (please specify): ______________ 4. Field of Study: _______________

*Section 1: Knowledge* 1. Have you heard about interdental aids? o ☐ Yes o ☐ No 2. What are interdental aids primarily used for? o ☐ Cleaning between teeth o ☐ Whitening teeth o ☐ Strengthening enamel o ☐ Other (please specify): _______________ 3. Which of the following are examples of interdental aids? (Select all that apply) o ☐ Dental floss o ☐ Interdental brushes o ☐ Water flossers o ☐ Toothpicks o ☐ None of the above 4. How often should interdental aids be used according to dental professionals? o ☐ Daily o ☐ Weekly o ☐ Occasionally o ☐ Not sure

*Section 2: Awareness* 1. Are you aware of the health benefits of using interdental aids? o ☐ Yes o ☐ No 2. Do you think interdental cleaning is as important as brushing your teeth? o ☐ Yes o ☐ No

3. Have you received any formal education or training about the use of interdental aids? o ☐ Yes (please specify source): ______________ o ☐ No

*Section 3: Attitude* 1. How important do you think it is to use interdental aids for oral health? o ☐ Very important o ☐ Somewhat important o ☐ Not important o ☐ Not sure 2. Would you recommend interdental aids to friends or family members? o ☐ Yes o ☐ No o ☐ Not sure 3. What prevents you from using interdental aids? (Select all that apply) o ☐ Lack of knowledge o ☐ Cost o ☐ Time-consuming o ☐ Difficulty in use o ☐ Other (please specify): _______________

*Section 4: Practices* 1. Do you currently use any interdental aids? o ☐ Yes (please specify): ______________ o ☐ No 2. How often do you use interdental aids? o ☐ Daily o ☐ A few times a week o ☐ Occasionally o ☐ Never 3. What type of interdental aid do you use most often? o ☐ Dental floss o ☐ Interdental brushes o ☐ Water flossers o ☐ Toothpicks o ☐ Other (please specify): ______________ 4. Where do you typically learn about oral hygiene practices? o ☐ Dentist o ☐ Social media o ☐ Friends/Family o ☐ Online articles/videos o ☐ Other (please specify): _______________ 5. Do you face any challenges in using interdental aids?

o ☐ Yes (please specify): ______________ o ☐ No
